# Rectal Surgery

**Published:** 1895-01-19

**Authors:** 


### RECTAL SURGERY.

**Fistula.**—Dr. John Platt[1] draws some instructive deductions from an analysis of seventy-six cases of fistula in ano under his care. He found the affection very much more common in males than females. With regard to cause nothing very definite could be obtained, the origin of many being quite obscure. He found, however, the proportion of phthisical cases greater than that usually given, perhaps because of the thoroughness of his examinations; the percentage in his cases was 28·7. He very truly remarks that no definite statement is to be found in text-books as to the duration of healing after operation. A careful record of his cases shows that phthisical fistulæ require a longer time than non-phthisical, the time required being on an average nine weeks and seven weeks respectively. He considers that if tuberculosis in the lungs is comparatively quiescent an operation is desirable. The general health of the patients with phthisis improved, and remained good for many months after the operation in every case; and the bad results said to occur after operation in phthisis were almost if not quite non-existent.

**Maglignant Growth of the Rectum.**—The same writer[2] has collected and completed the records of twenty-one cases of excision of the lower end of the rectum for cancer, performed in the Manchester Royal Infirmary since 1888. Out of the twenty-one cases there were three deaths from the operation, one from "prolonged shock," one from septic peritonitis, in which the peritoneum was accidentally opened, and one from septic periproctitis. The accidental opening of the peritoneum did not seriously complicate the operation, with the exception of the one fatal case. It happened five times, and in some of the cases the growth was low down in the rectum. Platt considers that the peritoneal wound should be sutured. In one case two wounds of the peritoneum were sutured, and although the patient died of septic periproctitis there was no peritonitis; and in another the peritoneal wound was not sutured, and the patient died from peritonitis. Stierlin is quoted as giving statistics of twenty-six cases in which the rent was sutured with twenty recoveries, and the same number of cases in which the opening was not sutured with sixteen recoveries.

In all the cases at the Manchester Infirmary, except three, the mucous membrane was brought down and sutured to the skin. Platt considers that this shortens the period of convalescence, and diminishes the tendency to subsequent stricture of the gut; but reference is made to Mr. Harrison Cripps' opinion that sutures cause a great deal of tension, that they are almost certain to cut their way out, and that their use greatly increases the risk of cellulitis and peritonitis from accumulation of septic fluids outside the bowel. Of course, sometimes the mucous membrane cannot be brought down to the skin. This was so in three cases out of the twenty-one in which it was not sutured. Volkman and Czerny recommend suturing. Statistic of permanent cure are given from other cases, the time being too short to do so from the Manchester Infirmary operations, but it is not stated whether the excision was performed by the perineal method or by Kraske's operation, in which a portion of the sacrum is removed. However, out of 375 cases, one in ten had no recurrence at the end of four years. These were mostly from German clinics. Kocher had five patients who were perfectly well after nine years.

The mortality after excision of the rectum is shown from statistics of a large number of cases to be from 14 to 20 per cent., the causes of death being chiefly periproctitis and peritonitis. Colotomy has a much smaller death-rate, but if the patient survive the operation of excision, life is more prolonged, and greater relief is given than after colotomy.

Removal of the coccyx often facilitated the removal of the growth, and did not add to the danger of the operation. It was removed four times, and with a small portion of the sacrum twice.

Nearly all the patients had very fair control over the bowels, except when the motions were liquid, and several had no incontinence whatever. In Mr. Harrison Cripps' patients the result in this respect has been much the same. The lower portion of the bowel was preserved in one of the cases at Manchester with a good result, and Platt discusses the advisability of preserving the sphincters and lowest portion of the bowel, and uniting the cut ends by means of sutures, in order to prevent incontinence. According to Arnd, in eight out of eighteen cases so treated the sutures cut out, and extravasation of fæces occurred. Von Bergman and Kocher are not in favour of such a proceeding.

The early age at which cancer of the rectum may occur is well illustrated by Platt's series of cases, for one patient was only nineteen, and four were under thirty-five. Mr. Swinford Edwards has seen two cases under twenty.[3]

Mr. Swinford Edwards calls attention[4] to the frequent mistake that is made in the diagnosis of cancer of the rectum at an early stage when the chance of removal is good. For want of digital examination in many cases piles are diagnosed. If cancer was discovered earlier, Mr. Edwards considers 75 per cent. would permit of removal, whereas now only 25 per cent. are favourable ones for extirpation. The obstruction to the venous circulation by the growth of the cancer, and the frequent straining during defæcation, may produce hæmorrhoids. Mr. Edwards has seen these secondary piles removed as the primary disease, the carcinoma remaining undiscovered. Mr. Edwards is in favour of colotomy as a preliminary measure to excision of the lower end of the rectum, in all cases where, owing to the extent of the disease, there is reason to fear the peritoneal cavity may be opened during the excision, whether perineal or acral.

The operation of excision of the portion of the rectum which cannot be reached from the perineum, by removal of the coccynx and part of the sacrum, was first introduced by Kraske,[5] and modified by various surgeons. As the operation is little known in this country, though it has been frequently performed in Germany, it may be as well to give a brief description of it before referring to recent communications on the subject. An incision is first made in the middle line, from the second sacral vertibra to the anus, dividing the muscular attachments to the sacrum as far as the end of the bone on the left side; the coccynx is then excised, the two sacro-sciatic ligaments detached from the sacrum, and a portion of the lower end of the sacrum cut away below the third sacral foramen, but not extending to the middle line. The bone is divided in a line beginning on the left edge at the level of the third posterior sacral foramen, and running in a curve concave to the left, and through the fourth foramen to the left lower corner of the sacrum. It is said that the nerves are avoided, and the sacral canal is not opened[6]. The rectum can be removed up to its junction with the sygmoid flexure. The operation has been modified in various ways by German and American surgeons. Bardenheuer advises transverse section of the bone below the third sacral foramen, and his operation does not seem to be followed by any material discomfort. Kammerer[7] has practised what he calls osteoplastic resection of the sacrum in six cases.

The operation was recommended by Rydygier, and consists in a transverse division of the sacrum below the third foramen, without division of the soft parts on the right side, the lower end of the bone with the coccyx being turned over in a hinge-like manner to the right, and replaced at the end of the operation. In the six cases in which Kammerer operated by this method, three were for rectal cancer. In the first case, the lower five inches of the rectum were removed; the upper end could be brought down and sewn to the margin of the wound at the site of the normal anus. Recovery was uninterrupted. Recurrence took place six months after, and was removed, and now has again occurred a year after. In the second case the patient seems to have died of shock. In the third, the central portion of the rectum was removed and the ends sutured, but the sutures gave way posteriorly and a fœcal fistula formed, and in two months' time the gut was found to be strictured at the junction. A secondary resection of the stricture was made, and the fœcal fistula thus closed. It was only possible to unite the two ends after resection of the stricture by raising the lower end, after making an incision in the perineum, to free the anus. Only in the third case was a preliminary inguinal colotomy performed. Dr. Rehn, of Frankfort,[8] operates in a similar manner to Dr. Kammerer with regard to the resection of the lower part of sacrum, but he operates in two stages. In the first, the rectum is brought up to the level of the skin by passing underneath it strips of iodoform gauze, and in the second, the growth is removed and the divided ends sutured. Murphy's button has been used to unite the divided end of the rectum in these sacral operations. The suggestion was made by Dr. Walker, of Detroit, and carried out by Dr. Marcy.[9] Four inches of the middle of the rectum were removed for carcinoma, and the ends united in this way. The button was removed on the twelfth day with a little difficulty, not having freed itself entirely from its attachment; but its removal was hastened because pressure of the button upon the neck of the bladder caused discomfort and some difficulty in micturition A constricting ring of cicatricial tissue was left, but convalesence was rapid and uneventful. When Murphy's button was not used—in case three of Kammerer's—it will be remembered a stricture formed.

The mortality of Kraske's operation has varied considerably in the practice of various operators. König, of Göttingen, has only had a mortality of 12 per cent. in recent cases, whereas of nineteen cases collected by Dr. Axel Inversen, of Copenhagen, eight died. Ball has adopted the transacral extirpation of cancer, the bone being divided through the fourth segment in four cases, in all of which a successful result was obtained.[10] In one case almost the entire rectum was removed, and the upper end brought out through the sacral opening. In another the lower end of the rectum was left, and the two ends were sutured; they gave way the first time the patient defæcated, but the fœcal fistula thus formed soon healed. Ball and Kammerer both consider this very likely to occur in cases of rectorraphy, because of the resistance to the onward passage of fæces from the sphincter ani. Out of Ball's thirteen cases of excision of the rectum, two died of septic peritonitis and one of shock, but none of Kraske's operations were fatal.

[1] Med. Chronicle, 1894, June, p. 167. [2] Ibid, 1894, Sept., p. 419 [3] Clinical Journal, 1894, May 30th, p. 81. [4] Ibid. [5] Annals of Surgery 1885, Vol. II., p. 415. [6] Ball on Diseases of the Rectum, 1894, p. 374, and Cooper and Edwards on Diseases of the Rectum, p. 216. [7] Medical Record, July 28, 1894, p. 97. [8] Annals of Surgery, 1890, Vol. II., p. 375. [9] Mathew's Quarterly Journal of Diseases of the Rectum, Jan., 1894, p. 80. [10] Loc. cit.

